# Supplementary material for: A novel D-peptide modulates DCLK1 gelsolin interactions, reducing PDAC tumor growth
Source: Sci Rep. 2025 Oct 14;15:35811. doi: 10.1038/s41598-025-19722-z (PMC12521541; doi:10.1038/s41598-025-19722-z)
Supplement: Supplementary file 1 — Supplementary Material 1 [file 41598_2025_19722_MOESM1_ESM.docx]

Supplementary Materials for

A Novel D-peptide modulates DCLK1 Gelsolin interactions, reducing PDAC tumor growth

**Landon L. Moore^1,2^, Dongfeng Qu^1^, Parthasarathy Chandrekesan**†**^1,3^, Kamille Pitts^1,2^, Randal May^1^, Byron E. Anderson^4^, Milton Brown^5^, and Courtney W. Houchen*^1,2,3^**

*Corresponding author, courtney-houchen@ouhsc.edu (C.W.H.). Tel.: +(405)-271-5428; Fax: +(405)-271-5803

Fig. S1.

**
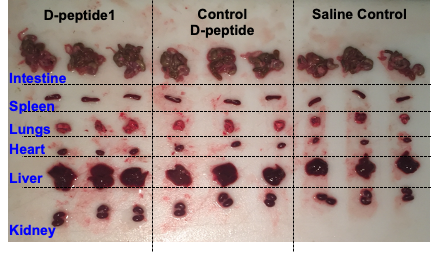
**

**Supplemental Figure 1. Assessment of DCLK1 D-peptide Toxicity in Mice.** Mice received escalating doses of D-peptide 1 every three days, starting with 750 µg (equivalent to 210 nM in blood and extravascular fluid) and increasing by 500 µg per dose, reaching a maximum of 4000 µg/mouse. Monitored toxicity indicators included soft feces, urine stains, depression, rough coat, nasal/ocular discharge, hunching, and body weight changes. Mice exhibited no adverse effects compared to saline-treated controls. Major organs were collected, and serum was stored for further biochemical analysis.

**Fig. S2.**

**
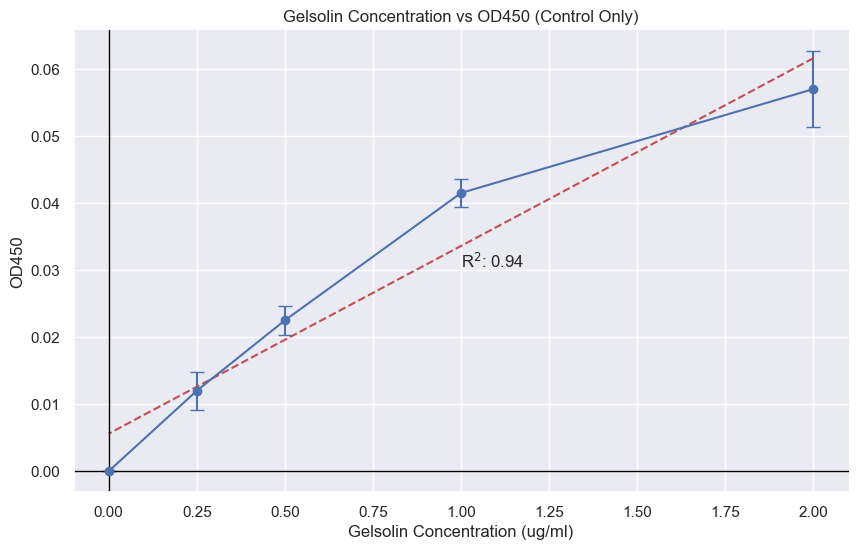
**

**Supplemental Figure 2. Plasma Gelsolin (pGSN) ELISA Binding Optimization Curve**

ELISA assays were performed where plates were coated with DCLK1 with increasing concentrations of plasma gelsolin were added and a dose curve based on Gelsolin protein was constructed. Peak concentration for pGSN was identified as 2 μg/ml with a R^2^ of 0.94.

Supplemental Table 1.

**Supplemental Table 1. SPR Analysis of DCLK1 isoform 4 with Gelsolin and FGF19**

| **Protein** | **MW (Da)** | **Kinetic 1:1**  **Ave K_a_ (M^-1^s^1^)** | **Kinetic 1:1**  **Ave K_d_ (s^1^)** | **Kinetic 1:1**  **Ave K_D_ (μM)** | **Steady-state Affinity Ave K_D_ (μM)** |
| --- | --- | --- | --- | --- | --- |
| FGF19 | 21430 | 5.09E+03 | 9.13E-03 | 1.794 | 0.970 |
| Gelsolin | 85710 | 5.48E+03 | 5.47E-04 | 0.099 | 1.760 |
